# SNCG promotes the progression and metastasis of high-grade serous ovarian cancer via targeting the PI3K/AKT signaling pathway

**DOI:** 10.1186/s13046-020-01589-9

**Published:** 2020-05-07

**Authors:** Jing Zhang, Xiao-han Liu, Cong Li, Xiao-xing Wu, Yan-lin Chen, Wen-wen Li, Xian Li, Fan Gong, Qin Tang, Dan Jiang

**Affiliations:** 1 Department of Obstetrics and Gynecology, the First Affiliated Hospital of Chongqing Medical University, Chongqing, 400016 China; 2 Department of Gastrointestinal Surgery, the First Affiliated Hospital of Chongqing Medical University, Chongqing, 400016 China; 3 Department of Pathology, Jinshan Hospital, the First Affiliated Hospital of Chongqing Medical University, Chongqing, 401122 China; 4 Department of Pathology, Faculty of Basic Medicine, Chongqing Medical University, Chongqing, 400016 China

**Keywords:** High-grade serous ovarian cancer, SNCG, Progression, Metastasis PI3K/Akt

## Abstract

**Background:**

The poor prognosis of patients with ovarian cancer is mainly due to cancer progression. γ-Synuclein (SNCG) has reported as a critical player in cancer metastasis. However, its biological roles and mechanism are yet incompletely understood in ovarian cancer, especially in high-grade serous ovarian cancer (HGSOC).

**Methods:**

This is a retrospective study of 312 patients with ovarian cancer at a single center between 2006 and 2016. Ovarian cancer tissues were stained by immunohistochemistry to analyze the relationship between SNCG expression and clinicopathologic factors. The clinical outcomes versus SNCG expression level were evaluated by Kaplan–Meier method and multiple Cox regression analysis. Next, systematical functional experiments were given to examine the proliferation and metastatic abilities of SNCG both in vitro and in vivo using loss- and gain- of function approaches. Furthermore, the mechanisms of SNCG overexpression were examined by human phospho-kinase array kit and western blot analysis.

**Results:**

Clinically, the expression of SNCG was significantly upregulated in ovarian cancer compared with the borderline and benign tumor, normal ovary, and fallopian tube. Notably, the high level of SNCG correlated with high-risk clinicopathologic features and showed poor survival for patients with HGSOC, indicating an independent prognostic factor for these patients. Functionally, we observed that overexpression of SNCG promoted cell proliferation, tumor formation, migration, and invasion both in vitro and in vivo. Mechanistically, we identified that SNCG promoted cancer cell metastasis through activating the PI3K/AKT signaling pathway.

**Conclusions:**

Our results reveal SNCG up-regulation contributes to the poor clinical outcome of patients with HGSOC and highlight the metastasis-promoting function of SNCG via activating the PI3K/Akt signaling pathway in HGSOC.

## Background

Ovarian cancer is the most lethal gynecological malignancy [1]. Globally, approximately 295,000 new cases and 184,000 deaths of ovarian cancer occur in 2018 [2]. More than 70% of patients are diagnosed with advanced disease (stage III/IV), which may be due to the absence of early symptoms and sensitive and specific biomarkers for early diagnosis. Histologically, the majority type of ovarian cancer is epithelial ovarian cancer (EOC). In recent years, EOC is recommended dividing into two subtypes due to distinctive morphologic and molecular genetic features [3]. Type I is composed of low-grade serous, endometrioid, clear cell, and mucinous carcinomas. Type II is mainly composed of high-grade serous ovarian cancer (HGSOC), which is the most aggressive behavior cancer and correlates with the poor clinical outcome as compared to other subtypes [4, 5]. Despite progress in treatment, the high rates of recurrence and metastasis correlate with poor prognosis of ovarian cancer [6]. Therefore, it is urgent to identify the novel targets and understand the mechanisms underlying ovarian cancer proliferation and metastasis.

γ-Synuclein (SNCG) is one member of the synuclein family (α-synuclein, β-synuclein, and SNCG), which was first named breast cancer-specific gene 1 (BCSG1) [7]. To date, the overexpression of SNCG has been demonstrated in multiple malignant solid tumors, including breast, ovarian, uterus, liver, and cervical cancers [8–10]. Besides, SNCG up-regulation is related to tumorigenesis and metastasis [10–12]. Studies suggest SNCG may be a potential prognostic marker and therapeutic approach to promote cancer progression, but the association of the SNCG overexpression with patient survival is controversial in EOC [13, 14]. Furthermore, one study has reported that SNCG might enhance the migration of ovarian cancer cells by activating small GTPases and ERKs of the RHO family [15]. However, the molecule mechanism of SNCG promoted ovarian cancer cell proliferation and metastasis is still not well understood.

Therefore, the present study aims to systematically examine the association of SNCG expression with clinicopathologic features and survival outcomes, the effect on biological functions both in vitro and in vivo, and the mechanism might involve in ovarian cancer progression and metastasis.

## Methods

### Patients and specimens

The 312 EOC tissues were obtained from the Department of Pathology, Chongqing Medical University, between January 2006 and September 2016. All patients underwent primary surgery and followed by cisplatin-based chemotherapy. No patients received neoadjuvant chemotherapy or radiation therapy. The borderline tumor tissues (*n* = 21), benign tumor tissues (*n* = 24), normal ovaries (*n* = 26), and fallopian tubes (*n* = 28, resected for non-ovarian diseases) were collected from patients who received gynecological surgeries in the First Affiliated Hospital of Chongqing Medical University between April 2014 and December 2016. All specimens were reevaluated for histological type and grade by three senior pathologists. The study protocols have been approved by the local Medical Ethics Committee, and the written informed consent was obtained from the patients.

### Cell lines and cell culture

Human ovarian tumor cell lines SKOV3, OVCAR3, OVCAR5, ES-2 were purchased from Keygen Biotech (Jiang Su, China). HO-8910, HO-8910 PM cell lines were purchased from Cell Bank of the Chinese Academy of Science (Shanghai, China). The HEY cell line was purchased from Shanghai Genechem (Shanghai, China). All cell lines were authenticated by profiling of short tandem repeat analysis. Cells were cultured in RPMI1640 (Invitrogen, Carlsbad, CA) supplemented with 10% fetal calf serum, penicillin (100 U/ml, Gibco) and streptomycin (100 μg/ml, Gibco). Cells were incubated in a humidified atmosphere containing 5% CO_2_ at 37 °C and checked regularly for mycoplasma infection.

### Reagents and antibodies

Antibodies against SNCG (ab55424), Phalloidin (ab176753), Ki-67 (ab15580), F-actin (ab112124) were obtained from Abcam (Cambridge, UK). Antibodies against mTOR (sc-517,464), p-mTOR (Sec2448, sc-293,133), p70S6 kinase (sc-8418), GAPDH (sc-47,724), β-Actin (sc-47,778), Cyclin D1 (sc-8396), MMP9 (sc-393,859, sc-21,733), Vimentin (sc-6260) were purchased from Santa Cruz (CA, USA). Antibodies against p-p70S6 kinase (Thr389, #97596), p-Akt (Sec473, #4060), Akt (#4961), MMP2 (#40994), E-cadherin (#14472), N-cadherin (#13116) were purchased from Cell Signaling Technology. Secondary antibodies, including HRP-labeled goat anti-mouse IgG (A0216), HRP-labeled goat anti-rabbit IgG (A0208), Cy3-labeled goat anti-rabbit IgG (P0183), were purchased from Beyotime (Shanghai, China). LY294002 (ab120243) was obtained from Abcam (Cambridge, UK), Puromycin (sc-205,821) was purchased from Santa Cruz (CA, USA). insulin-like growth factor 1 (IGF-1) (P5502) was purchased from Beyotime (Shanghai, China). DMSO (PYG0040) and DAPI (AR1177) were purchased from Bosterbio (Hubei, China).

### Immunohistochemical staining (IHC)

The paraffin-embedded blocks were cut at 4 μm and mounted on slides. Following the deparaffinization, rehydration, blocking endogenous peroxidase, and antigen retrieval, the tissues were incubated with antibodies overnight at 4 °C. After washing in PBS, the tissues were incubated with the secondary antibodies for 30 min at 37 °C. Finally, sections were stained with a DAB staining solution and counterstained with hematoxylin. Negative control slides were incubated with normal goat serum. The immunostains were scored based on the intensity and the percentage of stained cells as described previously. Scores of 0 to 4 were defined as low expression, whereas scores of 5 to 9 were defined as high expression [16].

### Confocal immunofluorescence microscopy

Cells were seeded on sterile glass coverslips at 37 °C overnight and then fixed with 4% paraformaldehyde for 10 min, followed by blocking with 10% normal goat serum at 37 °C for 30 min. Subsequently, cells were incubated with the SNCG, MMP9, Vimentin, and F-actin primary antibodies at 4 °C overnight. After washing, cells were incubated with the secondary antibodies for 1 h. The F-actin cytoskeleton was visualized by staining with Phalloidin for 30 min following DAPI nuclear staining for 5 min. All images were taken with a laser scanning confocal microscope.

### Western blot analysis

Cells were harvested and lysed with the lysis buffer (Beyotime, Jiangsu, China) and centrifuged for at 12,000 g for 10 min at 4 °C. Following extraction, the total proteins were determined with BSA protein assay kits (Beyotime, Jiangsu, China). Next, equal amounts of protein (40 μg) were loaded onto 6–12% sodium dodecyl sulfate-polyacrylamide gels and electrophoretically transferred to polyvinylidene fluoride membranes (Millipore, MA, USA). Membranes were blocked with 5% non-fat dry milk in TBS with 0.1% Tween-20 for 1 h at 37 °C and then incubated with primary antibodies overnight at 4 °C, followed by exposure to secondary antibodies for 1 h at 37 °C. The GAPDH and β-actin were used as the internal control. Protein bands were detected by enhanced chemiluminescence plus detection reagents (Beyotime, Jiangsu, China).

### Quantitative real-time PCR (qRT-PCR)

Total RNA was extracted using Trizol reagent (Takara, Japan), and a cDNA reverse transcription kit (Takara, Japan) was used to synthesize the first-strand DNA following the manufacturer’s instructions. Briefly, each well contained a reaction volume of 25 μl. And the reaction was carried out using SYBR Green Kit and a CFX96™ real-time PCR Detection System (BioRad, USA). The primers sequences are listed in Table S1. The 2^−△△Ct^ method was used to determine the mRNA expression levels, with GAPDH as an internal control.

### Lentivirus construction and infection of cell lines

To suppress SNCG expression, three small hairpin RNA (shRNA) sequences targeting SNCG were synthesized by Sangon Biotech, Shanghai, China (sh1: 5′-GAAGCAGCTGAGAAGACCAAG-3′, sh2:5′-TCATGTATGTGGGAGCCAA GA-3′, and sh3: 5′-CCAAGGAGAATGTTGTACAGA-3′), and a scrambled shRNA was synthesized for the negative control (Scr: 5′-TTCTCCGAACGTGTCA CGTAA-3′). The SNCG cDNA was amplified and subcloned into pcDNA3.1 to construct SNCG overexpression vectors (Ove), and an empty vector was used as the negative control (Ctrl). Sequences are listed in Table S2. Subsequently, stable cell lines silencing or overexpressing SNCG were generated using the lentivirus vector following the manufacturer’s protocols. Infected cells were treated with 2 μg/ml puromycin, and the puromycin resistant clones were isolated. The transfection efficiency was confirmed by Western blotting and qRT-PCR.

### Cell proliferation and colony formation assays

For cell viability assays, cells (500 per well) were seeded in 96-well plates over 24 h to 96 h. Briefly, 20 μl of MTT solution was added to each well. After incubation for 4 h at 37 °C, 150 μl of DMSO was added to each well. Finally, A450 was measured using a microplate reader. To analyze the colony formation, cells (200 per well) were seeded in 24-well plate for 2 weeks, and then colonies were fixed with 4% paraformaldehyde and stained with 0.5% crystal violet. The colony formation was also assessed using a two-layer soft agar assay. Cells (1× 10^3^ per well), mixed in with a 1.5 ml of 0.3% top agar solution in RPMI1640 medium containing 10% FBS, were plated over a solidified base agar layer (1.5 ml of 0.6% agar solution) in 6-well plates. After 3 weeks, colonies containing over 50 cells were counted.

### Cell migration and invasion assays

Transwell assays were used to evaluate cell migration and invasion. The 8-μm pores (BD Bioscience, CA, USA) coated with a 1:5 dilution of Matrigel (BD Bioscience, CA, USA) were used in the invasion assay. Meanwhile, membranes without Matrigel coating in the chambers were used in the migration assay. The protocols were the same for both assays. Cells (1 × 10^4^ per well), suspended in the medium containing 5% FBS, were seeded in the upper chambers. Next, the medium containing 10% FBS was added to the lower chambers as a chemoattractant. After a 24 h incubation at 37 °C, cells on the lower side were fixed with 4% paraformaldehyde, stained with 0.5% crystal violet, photographed and counted under a microscope. Besides, a wound-healing assay was used to assess cell migration ability. Cells were seeded into 6-wells (1 × 10^6^ per well) and cultured until 90% confluence. Subsequently, cells were scratched with a 200-μL sterile tip and washed with PBS twice to remove the detached cells. Cells were allowed to grow for 24 h in a serum-free medium. The wound margins were observed and photographed under a microscope.

### Tumor metastasis in vivo

To study the effect of SNCG on tumor growth in vivo, 2 × 10^6^ cells per mice, trypsinized and resuspended in PBS, were injected intraperitoneally into 6-week-old athymic female nude mice (*n* = 5/group). After five weeks, the mice were sacrificed and autopsied. Visible metastatic implants were harvested and weighed. Excised tumor tissues were formalin-fixed and paraffin-embedded for IHC subsequently. All animal experiments were approved by the Institution Animal Care Committee at Chongqing Medical University, Chongqing, China.

### Human phospho-kinase array

The human phospho-kinase array kit (ARY003B) was purchased from R&D Systems and performed based on the manufacturer’s protocol. Briefly, 1.0 mL of array buffer was firstly added to each well for 1 h at room temperature. After washing, SKOV3 (Scr/sh1) cell lysates (400 μg per well) was added to each well overnight in array membranes at 4 °C. And then membranes were washed and incubated with detection antibodies for 2 h at room temperature. Next, membranes were exposed to streptavidin-HRP for 30 min on a rocking platform. After washing, protein bands were detected by enhanced chemiluminescence for 1 min and exposed to film. The experiment was performed three times.

### Statistical analysis

Statistical analysis was done using IBM SPSS Statistics for Mac (Version 23.0, IBM, USA). Continuous data are presented as mean ± standard deviation (SD), and categorical data are expressed as frequencies. Pearson Chi-Square test was used to compare the correlations of SNCG expression and clinicopathological features. The student’s t-test was used for analyzing two-group differences. All experiments were carried out at least three independent times. The relationship between SNCG expression and patient survival was examined by the log-rank test with the Kaplan-Meier method. Multiple Cox regression analysis was used to identify independent risk factors associated with survival. All tests were two-tailed with *P* < 0.05 significance.

## Results

### SNCG is overexpressed in EOC tissue and correlated with poor prognosis in patients with HGSOC

In our cohort of patients, the mean age was 51.2 years (range, 15–86 years). 239 (76.6%) patients were diagnosed with advanced-stage (III/IV), while 73 (23.4%) patients had early-stage (I/II). Of all patients, 87.5% underwent primary surgery with optimal debulking. The majority of patients were HGSOC (69.2%), and others had low-grade serous (7.7%), endometroid (10.2%), mucinous (5.8%), and clear cell (7.1%) types. The clinicopathologic features are summarized in Table 1.

**Table 1:** Correlation between the SNCG expression and clinicopathological characteristics in 312 patients with EOC

Characteristics	No. Patients	SNCG Expression		*P* value
	(*n* = 312)	Low No. (%)	High No. (%)	
Age(years)				
< 51.0	169	58 (34.3%)	111 (65.7%)	
≥ 51.0	143	41 (28.7%)	102 (71.3%)	0.285
Ca125 (U/ml)				
< 105	53	26 (49.1%)	27 (50.9%)	
≥ 105	259	73 (28.2%)	186 (71.8%)	**0.003**
FIGO stage				
I/II	73	40 (54.8%)	33 (45.2%)	
III/IV	239	59 (24.7%)	180 (75.3%)	**< 0.001**
Grade				
1	58	26 (44.8%)	32 (55.2%)	
2/3	254	73 (28.7%)	181 (71.3%)	**0.018**
Histology				
Type I	96	48 (50.0%)	48 (50.0%)	
Type II	216	51 (23.6%)	165 (76.4%)	**< 0.001**
Ascites				
Yes	211	62 (29.4%)	149 (70.6%)	
No	101	37 (36.6%)	64 (63.4%)	0.198
Cytoreductive surgery				
Optimal	273	80 (29.3%)	193 (70.7%)	
Sub-optimal	39	19 (48.7%)	20 (51.3%)	**0.015**

**Fig. 1:**
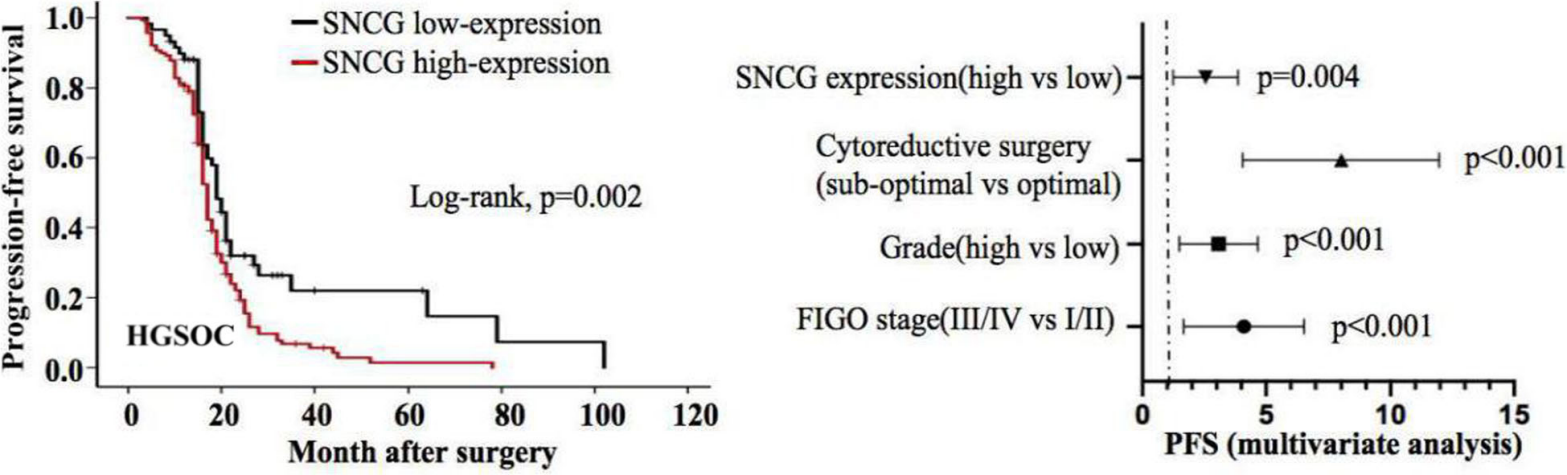
SNCG is overexpressed in EOC tissue and correlated with poor prognosis in patients with HGSOC. **a** IHC staining showed SNCG expression in the normal ovary, fallopian tube tissues, benign tumor, and borderline tumor (original magnification, × 200). **b** Representative images of IHC staining of SNCG expression in different pathological types of EOC tissues (original magnification, upper× 200, lower×400). **c** Kaplan-Meier analysis was performed for EOC patients to analyze the association between SNCG expression and survival outcome (OS and PFS). **d** In the HGSOC cohort, Kaplan-Meier analysis indicated the correlation of SNCG overexpression with OS (upper) and PFS (lower). By multivariate Cox regression analysis (Forest plot), results showed that SNCG overexpression was an independent prognostic factor

The high level of SNCG expression was observed in 68.3% of EOC tissues by IHC staining. The expression of SNCG was significantly increased in the EOC tissues compared with the borderline tumor, benign tumor, normal ovary, and fallopian tube. (Fig. 1a-b). Moreover, representative staining images of SNCG expressed in different pathological types were also shown in Fig. 1B. The association between the SNCG expression and clinicopathologic parameters of EOC was subsequently analyzed. Results showed that up-regulated SNCG positively correlated with the high CA125 values (*p* = 0.003), advanced stage (*p* < 0.001), high-grade (*p* = 0.018), HGSOC (p < 0.001), and suboptimal debulking tumors (*p* = 0.015), but not associated with age, tumor histology, or ascites (Table 1). Furthermore, we explored the relationship between SNCG expression and the survival time of EOC patients. The median progression-free survival (PFS) and overall survival (OS) were 19 months and 38 months, respectively. Kaplan-Meier analysis indicated a significantly worse PFS of patients in the SNCG-high expression group than those in the SNCG-low expression group (*p* = 0.007). However, the data failed to show a statistically significant correlation between SNCG expression and OS (*p* = 0.055, Fig. 1c). Patients harboring HGSOC mostly diagnose at advanced stages and have shorter progression-free survival. Thus, we hypothesized that overexpression of SNCG might be related to the survival of HGSOC. As expected, a significant correlation was identified between SNCG up-regulation and clinical outcome (PFS and OS) in patients with HGSOC. Moreover, multivariate analysis showed that SNCG was an independent prognostic factor for HGSOC patients (Fig. 1d).

### SNCG accelerates cell proliferation, facilitates cell migration and invasion in vitro

**Fig. 2:**
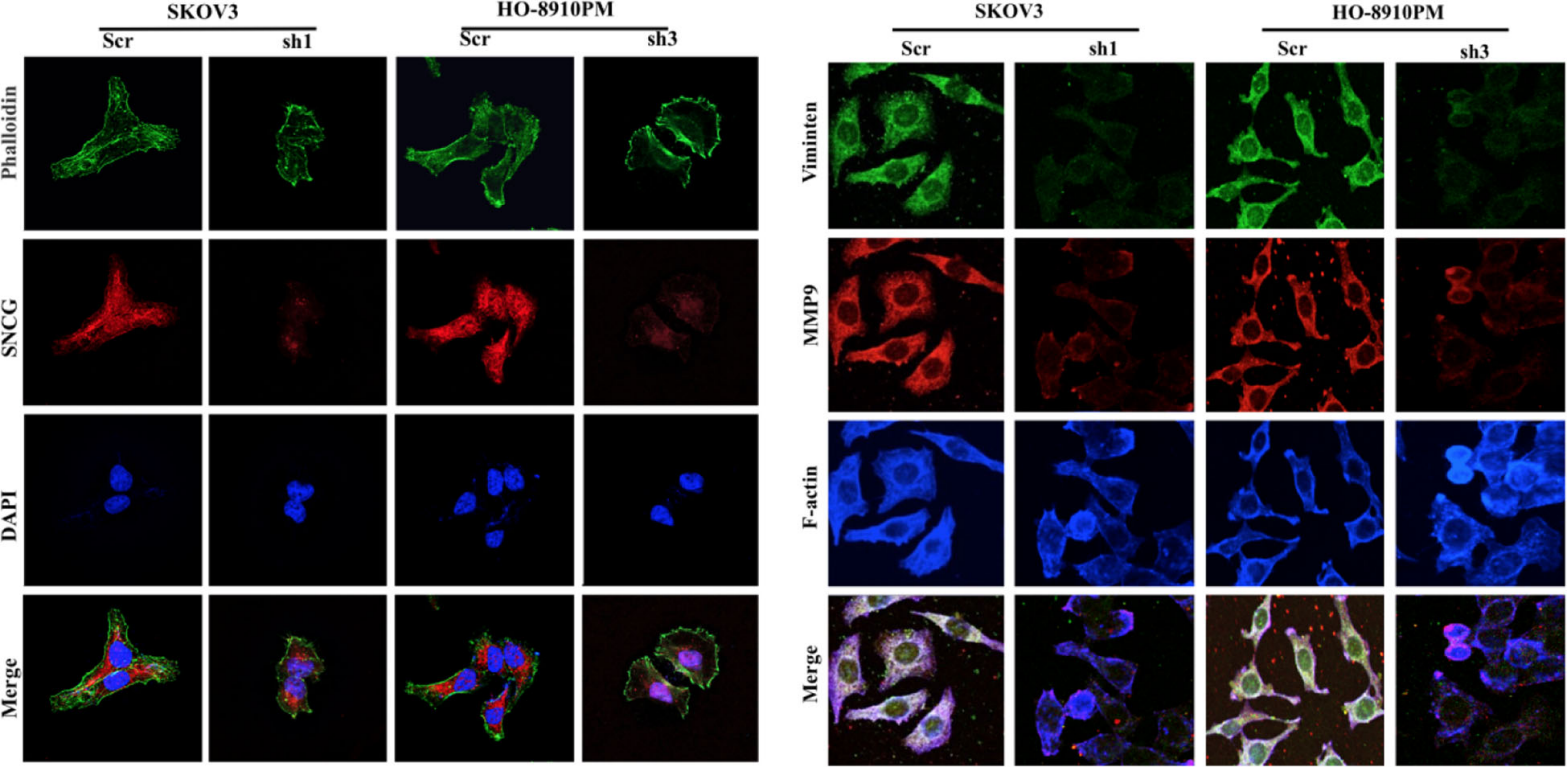
SNCG accelerates ovarian cancer cell proliferation, facilitates cell migration and invasion in vitro. **a** Western blot analysis of SNCG expression in different ovarian cancer cell lines. **b** The transfection efficiency was confirmed by Western blotting and qRT-PCR in SKOV3, HO-8901 PM, and OVCAR3 cells. **c** The MTT assay was used to detect ovarian cancer cell viability. **d** A soft agar assay was used to examine the proliferation of ovarian cancer cells. **e** Cell migration and invasion capabilities were determined using transwell assays (original magnification, × 200). **f** and **g** SKOV3 and HO-8910 PM cell transfectants were plated on FN and stained for SNCG, phalloidin, and nuclear. Moreover, cells were stained for Vimentin, MMP9, and F-actin. The individual or merged images visualized by a laser scanning confocal microscope (original magnification, × 1000). ^▲^, *P* < 0.05. *, *P* < 0.001. Ctrl: control; Ove: overexpression; Scr: scramble; sh1: small hairpin RNA 1; sh2: small hairpin RNA 2; sh3: small hairpin RNA 3

To determine the contribution of SNCG to the malignant behaviors of ovarian cancer cells, we first used Western blot to assess the endogenous expression of SNCG in different cell lines. The results showed that SNCG was highly expressed in SKOV3 and HO-8910 PM cells, whereas a low level of SNCG was detected in OVCAR3 (Fig. 2a). We chose these three kinds of HGSOC cell lines for further study. Next, we lentivirally introduced specific SNCG shRNA into SKOV3 and HO-8910 PM cells and successfully established stable cell lines (SKOV3-sh1 and HO-8910 PM-sh3, Fig. 2b). The MTT and colony formation assays showed that knockdown of SNCG suppressed the viability and colony formation in SKOV3-sh1 and HO-8910 PM-sh3 cells (Fig. 2c-d). Using transwell assays, we observed that the migratory and invasive capacities of SKOV3-sh1 and HO-8910 PM-sh3 cells were noticeably inhibited (Fig. 2e). Besides, we overexpressed SNCG in OVCAR3 cells (OVCAR3-Ove). As expected, the ectopic expression of SNCG significantly enhanced EOC cell proliferation and clonogenicity (Fig. 2c-d). Meanwhile, the transwell assay showed a substantial increase in cell migration and invasion of OVCAR3-Ove cells (Fig. 2e). The effect of SNCG on cancer cell morphology was then evaluated by F-actin cytoskeleton staining combined with FN exposure. We observed that the cell polarization and lamellipodia formation were limited in SNCG-knockdown cells compared with control cells. Furthermore, knockdown of SNCG reduced the expression levels of MMP9 and Vimentin (Fig. 2f and g). Thus, SNCG silencing inhibited the ovarian cancer cell migratory capacity, which might contribute to its metastatic inhibition in ovarian cancer.

### SNCG promotes cell growth and metastasis in vivo

**Fig. 3:**
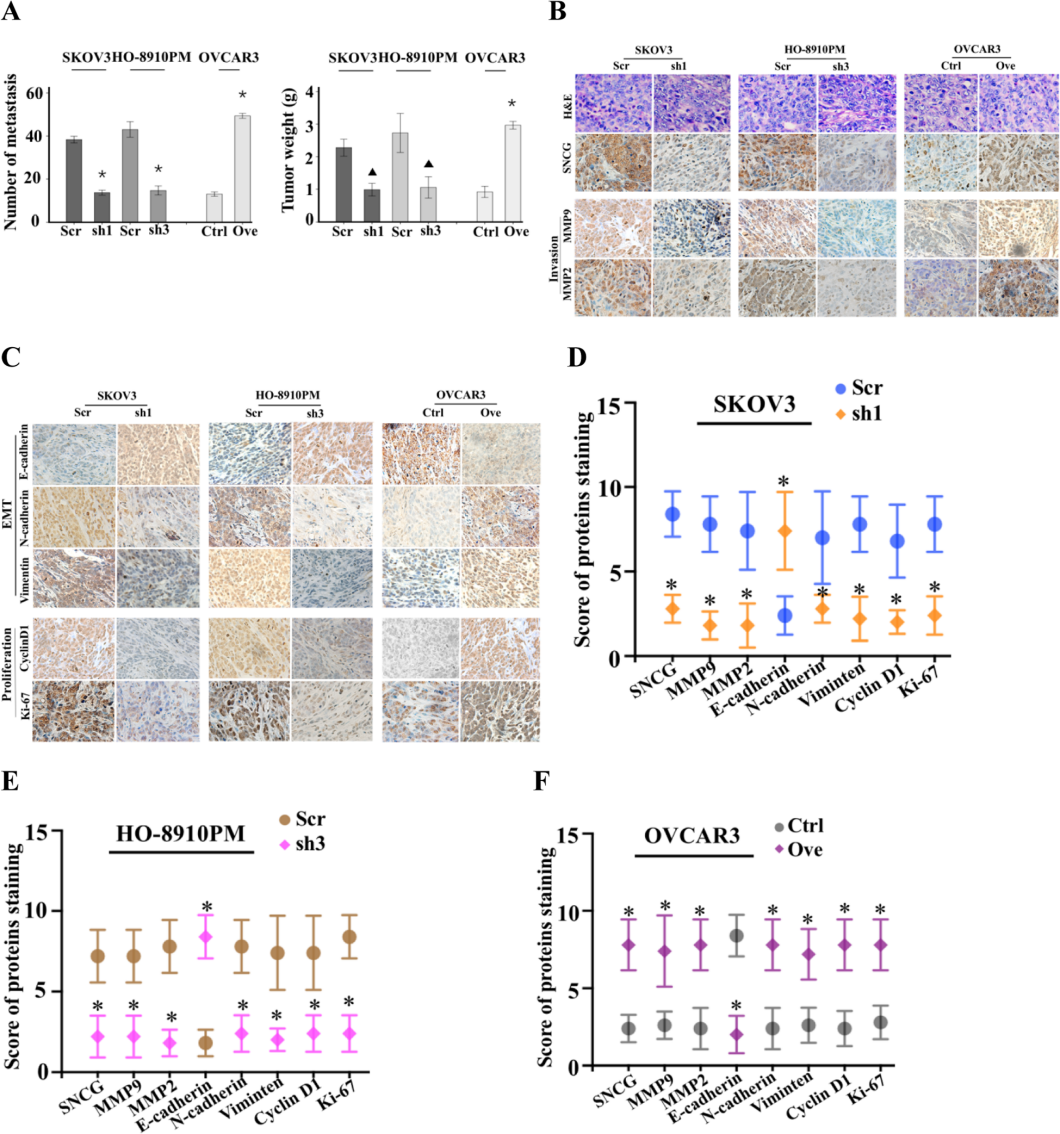
SNCG promotes ovarian cancaer cell growth and metastasis in vivo. **a** The number of tumor metastasis (left) and tumor weight (right) were collected and weighed. **b** and **c** The IHC staining was applied to assess the expression of SNCG, MMP9, MMP2, E-cadherin, N-cadherin, Vimentin, cyclin D, and Ki67 in xenograft tumor metastasis (original magnification, × 200). **d** and **e** The scores of IHC staining for proteins in the SNCG silencing groups compared with the Scr groups. **f** The scores of IHC staining for proteins in the OVCAR3-Ove group compared with the Ctrl group. ^▲^, *P* < 0.05. *, P < 0.001

To determine the potential impact of SNCG in cell growth and metastasis in vivo, we intraperitoneally injected SNCG knockdown (SKOV3-sh1 and HO-8910 PM-sh3) and overexpression (OVCAR3-Ove) cells into female athymic BALB/c nude mice to form peritoneal metastases. Mice were sacrificed after five weeks. Consistent with human disease, the overt metastatic lesions were attached to the mesentery, omentum, and liver. Compared with the control groups, both the number of metastatic nodules and tumor weight were significantly reduced in SNCG-knockdown groups. In contrast, the SNCG-overexpression group significantly increased the metastatic colonization and weight of implants (Fig. 3a). Next, tumor sections were stained to evaluate the proliferation, epithelial-mesenchymal transition (EMT) and metastasis-associated markers by IHC. As shown in Fig. 3b-f, we found that knockdown of SNCG was accompanied by reduced Ki-67, cyclin D1, MMP2, MMP9, N-cadherin, and Vimentin and increased E-cadherin immunostaining in metastatic tissues. Results were also supported in the SNCG overexpression group. Together, the data corroborated the in vitro results and provided evidence that SNCG was critical for the establishment of ovarian cancer metastasis in vivo.

### SNCG induces ovarian cancer progression through activating the PI3K/AKT signaling pathways

**Fig. 4:**
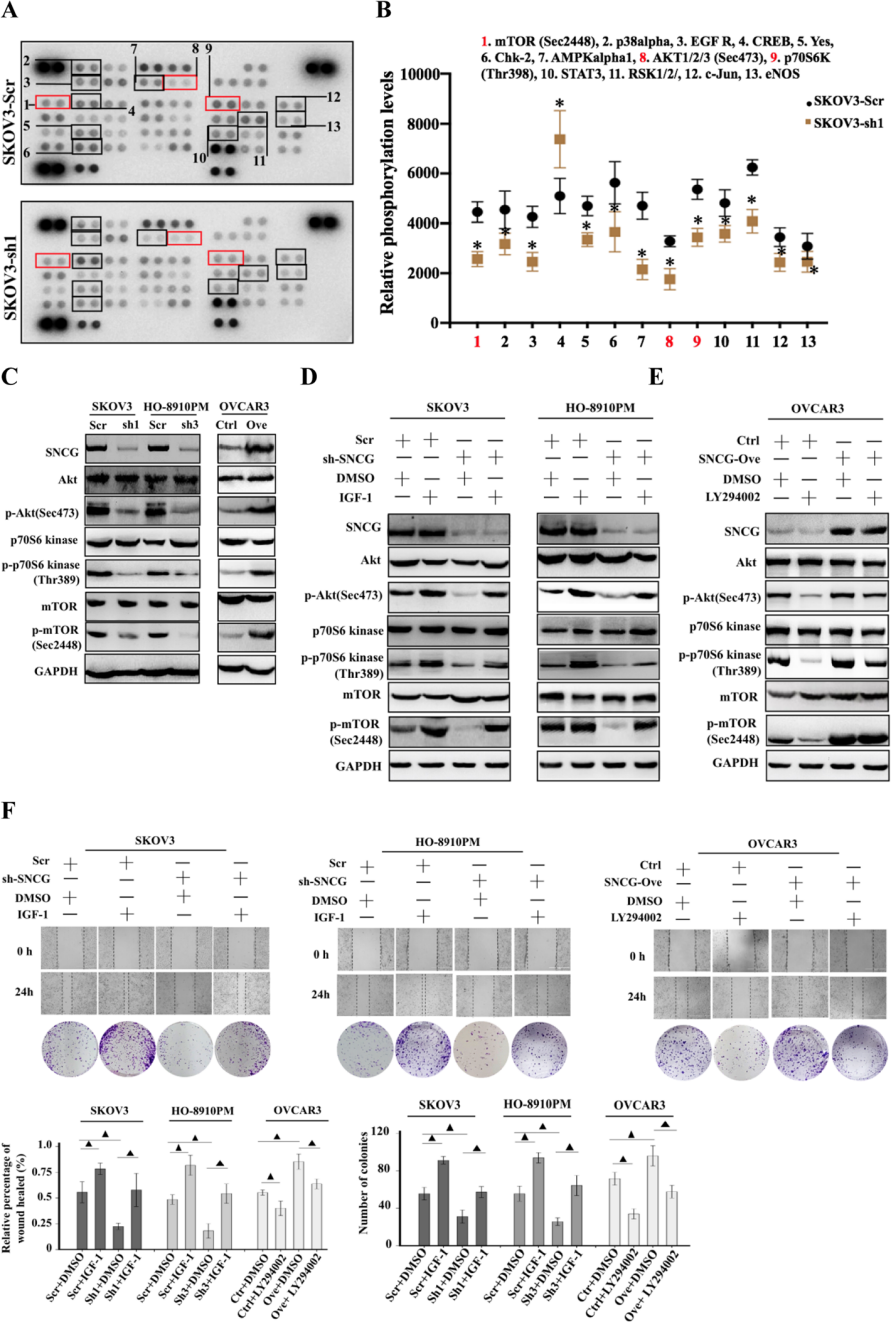
SNCG induces ovarian cancer progression via activating the PI3K/AKT signaling pathway. **a-b** A phospho-kinase array kit was performed on protein lysates of SKOV3-sh1 and control cells. Thirteen proteins were obvious changes in their phosphorylation status and highlighted by boxes. **c** Western blot analysis of the levels of Akt, p-Akt (Sec473), p70S6 kinase, p-p70S6 kinase (Thr389), mTOR, and p-mTOR (Sec2448) in SKOV3 and HO-8901 PM cells transfected with SNCG-shRNA or Scr, and OVCAR3 cells transfected with SNCG-Ove or Ctrl. **d** Expression levels of Akt, p-Akt (Sec473), p70S6 kinase, p-p70S6 kinase (Thr389), mTOR, and p-mTOR (Sec2448) in cells transfected with SNCG shRNA, Scr, SNCG Ove, Ctrl, IGF-1, and DMSO were determined by Western blot. **e** Expression levels of Akt, p-Akt (Sec473), p70S6 kinase, p-p70S6 kinase (Thr389), mTOR, and p-mTOR (Sec2448) in cells transfected with SNCG shRNA, Scr, SNCG Ove, Ctrl, LY294002, and DMSO were determined by Western blot. **f** Wound healing assay (upper, original magnification × 40) and cell colony formation (lower) of cells transfected with SNCG shRNA, Scr, SNCG Ove, Ctrl, IGF-1, LY294002, and DMSO confirmed the effect of SNCG on the PI3K/AKT signaling pathway. ^▲^, P < 0.05. *, *P* < 0.001. IGF-1: insulin-like growth factor 1; sh-SNCG: small hairpin RNAs of SNCG

Since loss- and gain-of-function of SNCG could regulate the aggressive behaviors of ovarian cancer cells both in vitro and in vivo, we tried to identify the underlying molecular mechanism. Thus, we performed a human phospho-kinase array, which is closely related to cell proliferation and migration. As shown in Fig. 4a-b, 13 kinases significantly changed after knockdown of SNCG in SKVO3 cells. The phosphorylation of kinases within the PI3K/Akt signaling pathway, including AKT1/2/3 (Sec473) and its downstream kinases p70S6K (Thr398), mTOR (Sec2448), was down-regulated in SKOV3-sh1 cells relative to control cells. These three kinases, members of the PI3K/Akt family, have been reported to be involved in the induction of EMT, which is a critical process in the pathogenesis of metastasis [17, 18]. Since recent studies indicate the role of SNCG on the PI3K/Akt signaling pathway in cancers [19, 20], we chose to further focus on the effect of SNCG on PI3K/Akt signaling pathway in HGSOC. To verify SNCG overexpression promoted EOC progression through regulating the PI3K/Akt pathway, we examined the phosphorylated proteins using western blot analysis. Our results showed that the expression levels of p-Akt, p-p70S6K, and p-mTOR were significantly decreased after knockdown of SNCG in SKOV3 and HO-8910 PM cells, and markedly increased after exogenous overexpression of SNCG in OVCAR3 cells (Fig. 4c). Furthermore, we observed that the IGF-1 (a PI3K agonist) could restore the expressions of p-Akt, p-p70S6K, and p-mTOR downregulated by knockdown of SNCG (Fig. 4d). In contrast, the high levels of these phosphorylated proteins could be decreased by LY294002 (a PI3K inhibitor) in SNCG ectopic-expressed cells (Fig. 4w). Moreover, the colony formation assay and the wound healing assay confirmed that the SNCG regulates cell proliferative and invasive abilities via the PI3K/Akt pathway in vitro (Fig. 4f). Overall, the PI3K/Akt signaling pathway activation is involved in SNCG-mediated promotion of HGSOC proliferation and metastasis.

## Discussion

Despite advances in radical surgery and chemotherapy, the majority of patients who suffered advanced HGSOC remain high metastasis and poor prognosis, leaving an urgent need for effective prevention and therapeutic measures [21–23]. Emerging evidence has indicated that SNCG is highly expressed and tightly associates with tumor progression and metastasis, including gynecologic processes (endometriosis, cervical, endometrial and ovarian cancers) [19, 24–26]. In the present study, we systematically investigated the expression, biological function, and mechanism of SNCG in HGSOC.

Our data showed that SNCG was significantly upregulated in EOC tissues and the overexpression of SNCG correlated with clinicopathological factors, including high CA125 level, HGSOC, high grade disease, advanced stage, and suboptimal debulking surgery. These findings were consistent with a previous study [13, 27]. Survival analysis revealed that increased SNCG expression correlated with worse prognosis in breast cancer and endometrial cancer [25, 28], yet the clinical outcomes of EOC were lacking and unclear. Strohl et al. found that SNCG overexpression did not correlate with patient survival, despite it was overexpressed in metastatic tumors and associated with high-risk clinical variables [13]. Additionally, Fekete et al. reported the increased expression of SNCG correlated to poor PFS with a lager datasets of EOC samples in a meta-analysis [14]. There are two histotypes of EOC (type I and type II), which show to be profoundly different diseases in terms of etiology, morphology, protein expression and molecular profile. Studying them together may be the reason for the unclear outcomes. In our cohort, we revealed that the higher level of SNCG had a significant correlation with worse PFS and OS in HGSOC patients, which indicated its role in HGSOC as well as a novel prognostic marker of HGSOC aggression. Some studies have shown that SNCG could be detected in the circulation of patients with cancer [29, 30]. Future studies are needed to evaluate the potential of serum SNCG levels as a biomarker for HGSOC patients. Collectively, our findings indicate that SNCG overexpression is associated with HGSOC progression as well as an important prognostic marker of survival in patients with HGSOC.

The cellular behaviors of SNCG have been demonstrated to promote tumor growth and invasion in the breast, gastric, and oral squamous cancers [11, 12, 31]. However, little research exists investigating the function of SNCG correlation with HGSOC. Pan et al. indicated that SNCG upregulation enhanced cell migration in breast and ovarian cancers using the Boyden chamber assay [26]. Thus, we performed a series of cellular functional experiments. Consistently, our findings revealed silencing of SNCG could significantly block cell proliferation, colony-forming, migration, and invasion. In contrast, overexpression of SNCG could enhance the malignant behaviors of ovarian cancer in vitro. The changes in the morphology and cytoskeletal reorganization of SKOV3 and HO-8910 PM supported the effect of SNCG on the process of cell invasion. Furthermore, the results collected from the nude mice (in vivo) supported and verified the ability of SNCG-mediated cell progression and metastasis. Together, these observations underscore the importance of SNCG in cell proliferation and metastatic potential.

**Fig. 5:**
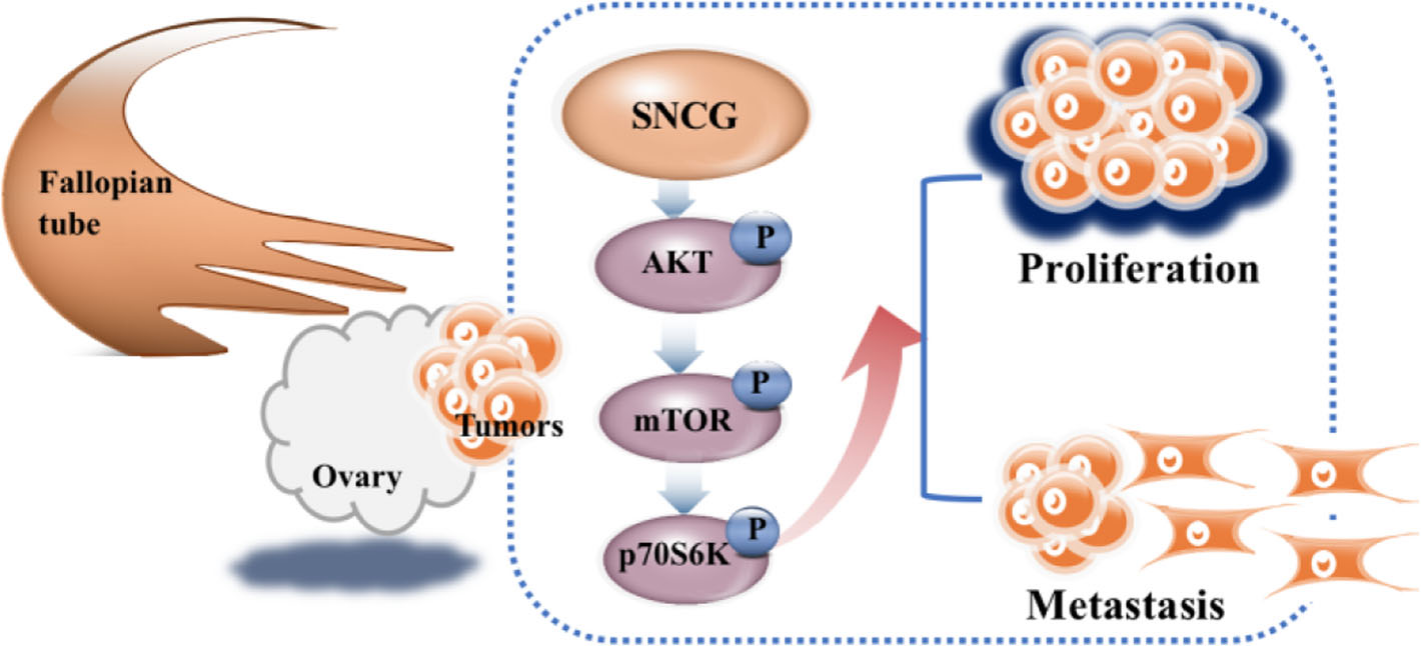
The schematic diagram summarizing the role of SNCG in promoting HGSOC progression. SNCG promotes the phosphorylation of AKT, mTOR, and p70S6K, which regulating the malignant behaviors in ovarian cancer cells

Recent studies show the overexpression of SNCG is tightly involved in the multiple complex mechanisms mediating tumor progression [10, 26, 31, 32]. Zhang et al. claimed SNCG could enhance tumor growth through the AKT pathway in cervical cancer [19]. Liang et al. reported that SNCG maintains pAKT might increase cancer progression and resistance to Hsp90 disruptors in breast cancer [20]. However, there is a lack of research on the mechanism of SNCG in the progression of ovarian cancer, especially in HGSOC. In our current study, we found SNCG could increase the phosphorylation of AKT and its downstream kinases, p70S6K, and mTOR. It is noteworthy that the PI3K/AKT pathway is an important activated signaling pathway and plays a critical role in multiple essential biological processes, such as cancer cell proliferation and invasion [33–36]. As expected, our data further indicated that SNCG overexpression could augment the activation of the PI3K/AKT pathway, while knockdown of SNCG could suppress this pathway. Using the PI3K inhibitor and agonist, we proved that the PI3K/AKT pathway was involved in SNCG promoting cell proliferation and metastasis. Overall, results suggest that SNCG induces ovarian cancer progression through regulating the PI3K/AKT signaling pathway (Fig. 5).

## Conclusions

The present study uncovered the high level of SNCG is a significant association with high-risk tumor features as well as an independent prognostic factor for HGSOC patients. Our systematic analysis showed the in vivo and in vitro effects of SNCG on ovarian cancer. Mechanistically, the up-regulated SNCG mediated cell proliferation and metastatic ability via activating the PI3K/AKT pathway of HGSOC. SNCG may serve as a potential therapeutic target and prognostic marker for HGSOC in the future.

## Supplementary information

**Additional file 1 Table S1**. The primers used in this study.

**Additional file 2 Table S2**. The cDNA sequence of SNCG.

## Data Availability

The data and materials of this study are available from the corresponding authors for reasonable requests.

## Abbreviations

BCSG1: Breast cancer-specific gene 1
BSA: Bovine serum albumin
Ctrl: Control
DAB: Diaminobenzidine
DAPI: 4′,6-diamidino-2-phenylindole
DMSO: Dimethyl sulfoxide
EMT: Epithelial-to-mesenchymal transition
EOC: Epithelial ovarian cancer
ERK: Extracellular regulated protein kinases
FBS: Fetal bovine serum
FIGO: International Federation of Gynecology and Obstetrics
GAPDH: glyceraldehyde-3-phosphate dehydrogenase
HGSOC: High-grade serous ovarian cancer
HRP: Horseradish peroxidase
IGF-1: Insulin-like growth factor 1
IHC: Immunohistochemical staining
MMP: Matrix metalloproteinase
MTT: 3-(4,5-dimethyl-2-thiazolyl)-2,5-diphenyl-2-H-tetrazolium bromide
OS: Overall survival; Ove: overexpression
PBS: Phosphate-buffered saline
PFS: Progression-free survival
PI3K: Phosphatidylinositol-3 kinase
qRT-PCR: Quantitative real-time PCR
Scr: Scramble
sh1: Small hairpin RNA 1
sh3: Small hairpin RNA 3
shRNA: Small hairpin RNA
SNCG: γ-Synuclein
