# Correction to: SNCG promotes the progression and metastasis of high-grade serous ovarian cancer via targeting the PI3K/AKT signaling pathway

**DOI:** 10.1186/s13046-020-01596-w

**Published:** 2020-06-17

**Authors:** Jing Zhang, Xiao-han Liu, Cong Li, Xiao-xing Wu, Yan-lin Chen, Wen-wen Li, Xian Li, Fan Gong, Qin Tang, Dan Jiang

**Affiliations:** 1 Department of Obstetrics and Gynecology, the First Affiliated Hospital of Chongqing Medical University, Chongqing, 400016 China; 2 Department of Gastrointestinal Surgery, the First Affiliated Hospital of Chongqing Medical University, Chongqing, 400016 China; 3 Department of Pathology, Jinshan Hospital, the First Affiliated Hospital of Chongqing Medical University, Chongqing, 401122 China; 4 Department of Pathology, Faculty of Basic Medicine, Chongqing Medical University, Chongqing, 400016 China

**Correction to: J Exp Clin Cancer Res (2020) 39:79**

**https://doi.org/10.1186/s13046-020-01589-9**

Following the publication of the original article [1], it was noted that due to a typesetting error the Figs. 1 and 2 were not complete. In addition, Fig. 1B should be changed to Fig. 1b, and Fig. 4w should be corrected to Fig. 4e in the main text of the article.

**Fig. 1:**
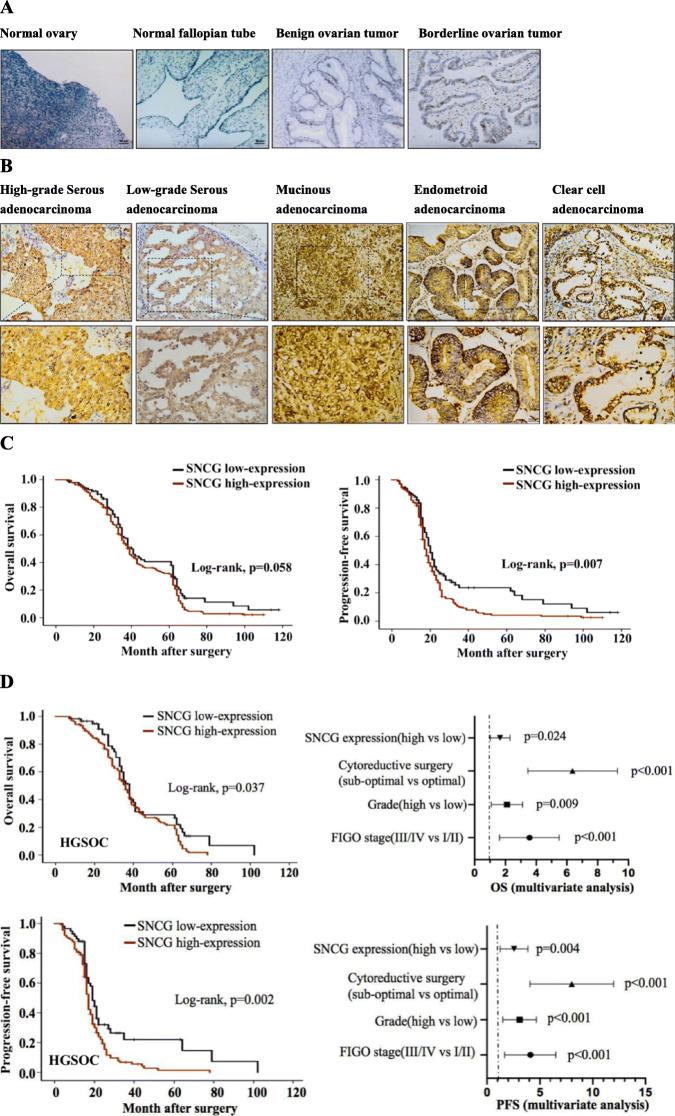
SNCG is overexpressed in EOC tissue and correlated with poor prognosis in patients with HGSOC. **a** IHC staining showed SNCG expression in the normal ovary, fallopian tube tissues, benign tumor, and borderline tumor (original magnification, × 200). **b** Representative images of IHC staining of SNCG expression in different pathological types of EOC tissues (original magnification, upper× 200, lower× 400). **c** Kaplan-Meier analysis was performed for EOC patients to analyze the association between SNCG expression and survival outcome (OS and PFS). **d** In the HGSOC cohort, Kaplan-Meier analysis indicated the correlation of SNCG overexpression with OS (upper) and PFS (lower). By multivariate Cox regression analysis (Forest plot), results showed that SNCG overexpression was an independent prognostic factor

**Fig. 2:**
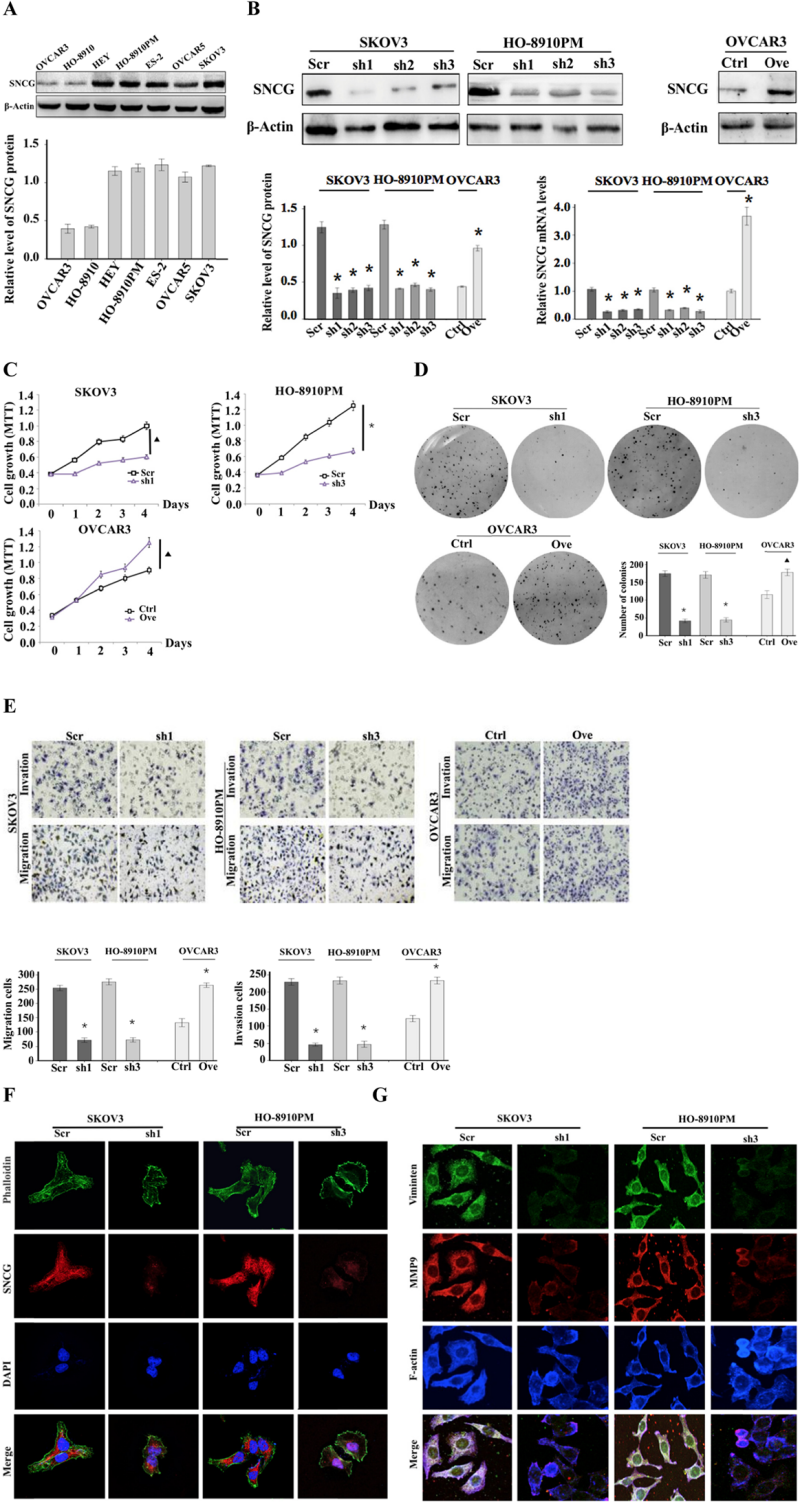
SNCG accelerates ovarian cancer cell proliferation, facilitates cell migration and invasion in vitro. **a** Western blot analysis of SNCG expression in different ovarian cancer cell lines. **b** The transfection efficiency was confirmed by Western blotting and qRT-PCR in SKOV3, HO-8901 PM, and OVCAR3 cells. **c** The MTT assay was used to detect ovarian cancer cell viability. **d** A soft agar assay was used to examine the proliferation of ovarian cancer cells. **e** Cell migration and invasion capabilities were determined using transwell assays (original magnification, × 200). **f** and **g** SKOV3 and HO-8910 PM cell transfectants were plated on FN and stained for SNCG, phalloidin, and nuclear. Moreover, cells were stained for Vimentin, MMP9, and F-actin. The individual or merged images visualized by a laser scanning confocal microscope (original magnification, × 1000). ^▲^, *P* < 0.05. *, *P* < 0.001. Ctrl: control; Ove: overexpression; Scr: scramble; sh1: small hairpin RNA 1; sh2: small hairpin RNA 2; sh3: small hairpin RNA 3

In the section “SNCG is overexpressed in EOC tissue and correlated with poor prognosis in patients with HGSOC”, (*p* = 0.055, Fig. 1c) should be corrected to (*p* = 0.058, Fig. 1c).

The correct figures have been included in this correction, and the original article has been corrected.
