# Supplementary material for: SNCG promotes the progression and metastasis of high-grade serous ovarian cancer via targeting the PI3K/AKT signaling pathway
Source: J Exp Clin Cancer Res. 2020 May 7;39:79. doi: 10.1186/s13046-020-01589-9 (PMC7204046; doi:10.1186/s13046-020-01589-9)
Supplement: Supplementary file 1 — Additional file 1 Table S1. The primers used in this study. [file 13046_2020_1589_MOESM1_ESM.docx]

| Supplementary Table 1. The primers used in this study | | |
| --- | --- | --- |
| Name | Primer Sequence (5’-3’) | Company |
| SNCG-F | ACCAAGGAGAATGTTGTACAGA | Sangon Biotech, Shanghai, China |
| SNCG-R | CTCTTTCTCTTTGGATGCCTCA | Sangon Biotech, Shanghai, China |
| GAPDH-F | GTATCGTGGAAGGACTCATGAC | Sangon Biotech, Shanghai, China |
| GAPDH-R | ACCACCTTCTTGATGTCATCAT | Sangon Biotech, Shanghai, China |
