# Supplementary material for: SNCG promotes the progression and metastasis of high-grade serous ovarian cancer via targeting the PI3K/AKT signaling pathway
Source: J Exp Clin Cancer Res. 2020 May 7;39:79. doi: 10.1186/s13046-020-01589-9 (PMC7204046; doi:10.1186/s13046-020-01589-9)
Supplement: Supplementary file 2 — Additional file 2 Table S2. The cDNA sequence of SNCG. [file 13046_2020_1589_MOESM2_ESM.docx]

| Supplementary Table 2. The cDNA sequence of SNCG | | |
| --- | --- | --- |
| Name | Sequence (5’-3’) | Company |
| SNCG | ATGGATGTCTTCAAGAAGGGCTTCTCCATCGCCAAGGAGGGCGTGGTGGGTGCGGTGGAAAAGACCAAGCAGGGGGTGACGGAAGCAGCTGAGAAGACCAAGGAGGGGGTCATGTATGTGGGAGCCAAGACCAAGGAGAATGTTGTACAGAGCGTGACCTCAGTGGCCGAGAAGACCAAGGAGCAGGCCAACGCCGTGAGCGAGGCTGTGGTGAGCAGCGTCAACACTGTGGCCACCAAGACCGTGGAGGAGGCGGAGAACATCGCGGTCACCTCCGGGGTGGTGCGCAAGGAGGACTTGAGGCCATCTGCCCCCCAACAGGAGGGTGAGGCATCCAAAGAGAAAGAGGAAGTGGCAGAGGAGGCCCAGAGTGGGGGAGACTAG | WanleiBio, Shenyang, China |
